# The Improved Physical Activity Index for Measuring Physical Activity in EPIC Germany

**DOI:** 10.1371/journal.pone.0092005

**Published:** 2014-03-18

**Authors:** Angelika Wientzek, Matthäus Vigl, Karen Steindorf, Boris Brühmann, Manuela M. Bergmann, Ulrich Harttig, Verena Katzke, Rudolf Kaaks, Heiner Boeing

**Affiliations:** 1 Department of Epidemiology, German Institute of Human Nutrition Potsdam-Rehbruecke, Nuthetal, Germany; 2 Division of Preventive Oncology (G110), National Center for Tumor Diseases (NCT), German Cancer Research Center (DKFZ), Heidelberg, Germany; 3 Division of Cancer Epidemiology (C020), German Cancer Research Center (DKFZ), Heidelberg, Germany; Universidad Pablo de Olavide, Centro Andaluz de Biología del Desarrollo-CSIC, Spain

## Abstract

In the European Investigation into Cancer and Nutrition study (EPIC), physical activity (PA) has been indexed as a cross-tabulation between PA at work and recreational activity. As the proportion of non-working participants increases, other categorization strategies are needed. Therefore, our aim was to develop a valid PA index for this population, which will also be able to express PA continuously. In the German EPIC centers Potsdam and Heidelberg, a clustered sample of 3,766 participants was re-invited to the study center. 1,615 participants agreed to participate and 1,344 participants were finally included in this study. PA was measured by questionnaires on defined activities and a 7-day combined heart rate and acceleration sensor. In a training sample of 433 participants, the Improved Physical Activity Index (IPAI) was developed. Its performance was evaluated in a validation sample of 911 participants and compared with the Cambridge Index and the Total PA Index. The IPAI consists of items covering five areas including PA at work, sport, cycling, television viewing, and computer use. The correlations of the IPAI with accelerometer counts in the training and validation sample ranged r = 0.40–0.43 and with physical activity energy expenditure (PAEE) r = 0.33–0.40 and were higher than for the Cambridge Index and the Total PA Index previously applied in EPIC. In non-working participants the IPAI showed higher correlations than the Cambridge Index and the Total PA Index, with r = 0.34 for accelerometer counts and r = 0.29 for PAEE. In conclusion, we developed a valid physical activity index which is able to express PA continuously as well as to categorize participants according to their PA level. In populations with increasing rates of non-working people the performance of the IPAI is better than the established indices used in EPIC.

## Introduction

The measurement of physical activity (PA) is a frequent challenge when designing an epidemiological study. Self-reported PA via questionnaire has long been the method of choice since such instruments are considered to be a cheap and easy way to assess PA. In practice, there is a plethora of questionnaires trying to estimate the amount of an individual's PA [1].

In the baseline examination of the European Investigation into Cancer and Nutrition study (EPIC) the EPIC Physical Activity Questionnaire (EPIC-PAQ 2) has been implemented to measure PA of the last 12 months [2]. Recently, also the Recent Physical Activity Questionnaire (RPAQ) has been applied in some EPIC subgroups [3], which covers a time frame of one month. Both questionnaires enable the calculation of PA indices: the Cambridge Index and the Total Physical Activity Index. Those were developed for adult populations and consider PA at work as a high impact item by cross-classifying the recreational activity level with the occupational activity. Furthermore, sedentary activities are not taken into account in these indices.

A recent validation study in the EPIC cohort for the RPAQ questionnaire showed fair validity for the Cambridge Index and for ranking habitual physical activity of individuals across European populations. Less satisfying results were shown for the Total Physical Activity Index [3].

Nowadays, objective measurements of PA like accelerometers are used more frequently. They provide continuous measures of PA and use acceleration transformed into counts to describe the amount of movement of a person [4]. A PA index that combines the advantages of a questionnaire and an accelerometer would provide the opportunity to describe PA in a new dimension.

Taking all the considerations into account, we aimed to develop an improved physical activity index, which is able to categorize study participants into activity categories but may also be used as a continuous measure that reflects PA and sedentary time. It is planned to accomplish these aims by analyzing the data from an extensive physical activity questionnaire and a 7-day heart rate and accelerometer based PA measurement obtained from a sub-sample of older adults form EPIC-Germany.

## Study Population and Instruments

### The EPIC-sub study

In the two German EPIC centers (Potsdam and Heidelberg) a sub-sample of 3,766 participants were selected from the original cohort. The selection process included information on age and sex in order to equally distribute the participants over the age and sex groups. 1,615 agreed to participate in the sub-study making up 3% of the original cohort of 53,088 participants, that were recruited at baseline from 1994 to 1998 from the general population of Potsdam and Heidelberg and surrounding communities [5], [6]. The validation sub-study was approved by the local ethics committees in Potsdam and Heidelberg (Ethics Committee of Medical Society of the federal state of Brandenburg (Potsdam) and the medical faculty of the University of Heidelberg). All participants, who signed an informed consent, were examined, asked about their disease history and informed by the physician prior to their inclusion in the study. Participants completed an extensive PA questionnaire and were also equipped with a combined heart rate and movement sensor that objectively measured PA continuously over a 7 day period. A simple step test was used for individual calibration of the heart rate response to exercise. Exclusion criteria for the PA part were physical impairment and plaster allergy. Step test exclusion criteria were severe cardiologic illness (i.e. aortic aneurysm, recent myocardial infarction, heart failure, myocarditis, cardiac dysrhythmia, cardiomyopathy, stroke, hypertension, and angina pectoris), current use of a beta blocker with more than half of the maximum dose/day, or physical disability preventing participants to walk unaided for a minimum of 10 minutes. If a smaller dose of beta blockers was used by the participant, a shorter version of the step test was performed.

We excluded participants that had less than 72 h of PA measurement (n = 159) or more than 20% of the measured data was lost due to measurement signal noise (n = 7). Furthermore, participants with incomplete or implausible questionnaire data were excluded (n = 105). Thus, 1,344 participants were available for analysis. For the split-sample internal validation we divided the sample. The training sample consisted of the first 433 participants (32%) (206 from Heidelberg and 227 from Potsdam) that attended the study centers. The validation sample included the remaining 911 (68%) participants (482 from Heidelberg and 429 from Potsdam).

### The Physical Activity Questionnaires

In the sub-study, several PA questionnaires were included. First, EPIC-PAQ 2 was applied [7]. It comprises questions about PA during the last 12 months including employment status, participation in several activities (walking, cycling, “do-it-yourself” activities, gardening, sports and household chores) separately for summer and winter, participation in vigorous non-occupational activities, and the average number of stairs climbed per day [7]. Second, the recently validated questionnaire RPAQ was applied. It asks about PA patterns in and around the house, travel to work, activity at work, and recreational activities during the last 4 weeks [3]. The Total PA Index and the Cambridge Index can be used for both questionnaires to assign participants to one of four PA categories (inactive, moderately inactive, moderately active and active).

The third questionnaire applied was the short ALPHA environmental questionnaire [8], which contains 11 questions about the residential environment. Lastly, we included three questions about the interest in sport activities and reasons of performing or not performing them based on the Trans-theoretical Model and Self Determination Theory. All answers were coded into absolute numeric character variables.

### The Actiheart

We used a validated combined accelerometer and heart rate monitoring device (Actiheart, CamNtech, Cambridge, UK), which was attached to the chest via two standard ECG electrodes [9].

Acceleration is measured by a piezoelectric element with a frequency sensitivity of 1–7 Hz (3 dB) and sampling frequency of 32 Hz. The Actiheart Software processes raw acceleration signals into a number of variables such as acceleration counts, total energy expenditure, physical activity energy expenditure (PAEE), physical activity level (PAL), and time spent in different activity intensity categories. The Actiheart is waterproof, which enables a continuous wearing period.

Before the Actiheart was initialized for long term recording a simple step test was performed in order to individually calibrate the physical activity intensity – heart rate relationship for each participant. The participants were asked to step up and down following a recorded voice that progressively speeded up from 60 steps/minute to 132 steps/minute across the 8 minutes of the test. When the participants' heart rate exceeded 85% of his/her age-dependent maximal heart rate or subjective, severe exhaustion symptoms occurred, the test was stopped [10]. A 2 minute recovery phase followed the test. Parameters from the step test were used to individually calibrate the relationship between heart rate and physical work load. If a participant was excluded from the step test or <4 min of step test data were available, age and sex dependent group calibration parameters were applied [11].

After calibration participants were requested to wear the device continuously for 7 days and send it back to the respective study center after this period. Data collected by the sensor were trimmed in each center applying the start- and end time point and calculating the mean sleeping heart rate from the mean heart rate from every night period (00:00–06:00) during the measurement. Step test data were centrally cleaned in Potsdam and the appropriate calibration model, as described above, was applied. Accelerometer counts [counts/min/day], PAEE [kJ/kg/day], moderate and vigorous physical activity (MVPA) [min/day] (>3 MET), time spent sedentary (<1.5 MET) [min/day] and the PAL were calculated for each individual by the Actiheart Software.

## Statistical Methods

First, Student's t and Chi-squared tests were applied to test for significant differences between the training sample and the validation sample in age, sex and objectively measured PA variables.

We calculated the Total Physical Activity Index and the Cambridge Index according to the recommendations [2], [12], [13]. Briefly, for the Total Physical Activity Index we summed up the time spent in different activities multiplied by their assigned metabolic equivalents (MET) values: for walking and housework (3.0 MET), for gardening (4.0 MET), for home repair (do-it-yourself work) (4.5 MET), for cycling and sports (6 MET), and for stair climbing (8.0 MET). The categorical level of occupational activity (sedentary/standing/manual/heavy manual/non-working) was cross-tabulated with combined recreational and household activities (in sex-specific quartiles of MET-hours/week). The Cambridge Index was built by the sum of average hours spent in cycling and sports during summer and winter and was also cross tabulated with categories of PA at work.

The selection of the questions for the Improved Physical Activity Index (IPAI) was performed in the following steps.

1. The univariate associations between the question from the RPAQ, EPIC-PAQ 2 and ALPHA questionnaire and the objective PA measures (acceleration counts, PAEE, PAL) were analyzed using Spearman correlation coefficients.
2. Subsequently, different approaches were used to combine the approximately 120 different sport variables to a reduced number of meaningful scores of sport activity. For the sport score (sport_score) we summed up all frequency of sport questions which were positively or negatively correlated with r ≥ ±0.05 with at least two of the objective activity variables, for the duration score (week4_sport) by summing up all positively correlated durations of sport questions; and for the intensity score (MET_sport) by adding all EPIC-PAQ sport intensity questions multiplied by the MET value of the particular sport according to the Compendium of Physical Activities [14].
  We summed up the positively correlated residential environmental variables and subtracted the negatively correlated variables to build a residential area score (residency_area).
  Sport_score =  sport^1^_freq_ + sport^2^_freq_ …+ sport^n^_freq_ if r>−0.05 between sport^n^_freq_ and at least 2 of the variables acceleration counts, PAEE, or PAL.
  Week4_sport =  sport^1^_dur_ + sport^2^_dur_ …+ sport^n^_dur_ if r>−0.05 between sport^n^_dur_ and at least 2 of the variables acceleration counts, PAEE, or PAL.
  MET_sport =  (sport^1^_int_ *MET^1^_sport_) + (sport^2^_int_ *MET^2^_sport_) …+ (sport^n^_int_ *MET^n^_sport_) if r>−0.05 between sport^n^_int_ and at least 2 of the variables acceleration counts, PAEE, or PAL.
  Residency_area =  resid_1_ + resid2 + resid_n_ – resid_n._
  Scores were also assigned for questions concerning television viewing and computer use frequency (television viewing and PC use categories: 1: never; 2: <1 h/day; 3: 1–2 h/day; 4: 2–3 h/day; 5: 3–4 h/day; 6: >4 h/day) and weighted by the number of weekdays (5/7 days  = 0.71) and weekend days (2/7 days  = 0.29):
  TV score  =  (TV weekday day + TV weekday evening) * 0.71+(TV weekend day +TV weekend evening) * 0.29;
  PC score  =  (PC weekday day +PC weekday evening) * 0.71+ (PC weekend day +PC weekend evening) * 0.29;
3. In a next step, Spearman correlation analyses were performed once more, including the newly generated scores. The obtained correlation matrix allowed choosing the new build sport variables with the highest correlation coefficient for accelerometer counts and also comparing the newly built television and computer use scores with each of the single television and computer use questions. This approach resulted in choosing the highest correlated sport, television, and computer use variables for further analyses.

Linear regression with stepwise selection, including the chosen sport, TV and PC variables from the last step, the residential environment variable and all remaining EPIC-PAQ, RPAQ questions, was used to define the final set of variables for the index that explains the most variance in accelerometer counts. For the resulting variables, descriptive distribution characteristics were calculated. In order to make the variables equally contribute to the final score of the index, they were standardized by their range (Variable value/Variable_range_) in the index formula. The index items were summed up following the direction they were correlated with accelerometer counts.

We calculated the IPAI score for every participant and ascribed 5 IPAI activity levels: inactive (<20th percentile), moderately inactive (20th–40th percentile), moderately active (40th–60th percentile), active (60th–90th percentile) and very active (>90th percentile).

The last step in the development process was the comparison of the IPAI and the two existing indices, Cambridge Index and Total Physical Activity Index by Spearman correlation coefficients with confidence interval estimation by Fishers z-transformation.

The index was further internally evaluated in the validation sample from the remaining study population. A sensitivity analysis with non-working participants (n = 699) was performed using Spearman correlations. Finally, we performed a linear regression analysis between the IPAI and accelerometer counts in order to determine β for future measurement error corrections analyses [15].

All analyses were performed in SAS Enterprise Guide version 4.3 SAS (release 9.2, SAS Institute, Cary, NC, USA).

## Results

Descriptive analysis showed no significant differences between the training and validation sample. Participants were on average 65 years old, accumulated 8.4 kJ/kg/day of PAEE, and a mean of 29.5 counts/min of accelerometer counts (Table 1).

**Table 1:** Study population characteristics.

		Training sample n = 433	Validation sample n = 911
Sex (%women)		50.81	51.59
Age at recruitment (years)		63.53±8.55	65.74±7.99
**Occupational activity (%)**	**Non-working**	51.27	52.58
	**Sedentary**	32.33	31.28
	**Standing**	9.70	10.43
	**Manual**	6.24	4.39
	**Heavy manual**	0.46	1.32
**Total Physical Activity Index (%)**	**Inactive**	20.55	20.42
	**Moderately inactive**	31.18	35.78
	**Moderately active**	42.26	38.20
	**Active**	6.0	5.60
**Cambridge index (%)**	**Inactive**	8.08	9.33
	**Moderately inactive**	35.80	34.25
	**Moderately active**	28.41	28.10
	**Active**	27.71	28.32
**Acceleration counts (counts/min/day)**		30.47±17.26	29.09±13.94
**PAEE (kJ/kg/day)**		8.54±3.66	8.29±3.68
**Sedentary time (min/day)**		1070±135	1077±136
**PAL**		1.59±0.20	1.58±0.20

The Spearman correlation coefficients between the set of RPAQ questions about the frequency of particular sports and the objective measures (accelerometer counts, PAEE, PAL) showed positive correlations (r>0.1) for nordic walking, cycling, high impact aerobics, exercises with weights, floor exercises, dancing, jogging, inline skating, and alpine skiing. Negatively correlated with at least two objectively measured variables were swimming, swimming-competitive, gardening, “do-it-yourself” activities, table tennis, and horse-riding. Two questions about the residency area from the ALPHA questionnaire (walking/cycling unsafe due to road traffic and high crime rate) showed negative correlations with the objectively measured PA. The remaining questions were positively correlated or showed no correlation.

Spearman correlation analyses with the newly formed scores revealed that the sport score (r = 0.33), PC weekend evening (r = 0.12), and the TV score (r = −0.31) were the variables that were highest correlated with accelerometer counts and were therefore included in the next step.

Multiple linear regression with stepwise variable selection gave a set of five variables explaining most of the variance: TV score (partial R = 0.086), sport score (partial R = 0.06), type of work (partial R = 0.02), cycling (h/week) (partial R = 0.01), and PC use weekend evening (partial R = 0.001). The model explained 18% of the variance in accelerometer counts. For the chosen variables univariate analyses were performed and the variable range was determined (Table 2).

**Table 2:** Characteristics for the Improved Physical Activity Index (IPAI) variables sport score, cycling (h/week), type of work, television viewing score, and weekend evening computer use in 433 participants of the EPIC Germany study training sample.

Variable	Mean	Median	SD	Min	Max	Range
**Sport score**	7.44	7	5.68	0	30	30
**Cycling**	2.26	1	3.19	0	20	20
**Type of work**	0.72	0	0.91	0	4	4
**TV score**	5.58	5.29	1.70	1.3	12	11
**PC weekend evening**	1.87	2	0.96	1	6	5

Variables were standardized by their range to account for the differences in the variable ranges and to make the variables equal contributing to the index.

Finally, for every participant, the index items were summed up: 
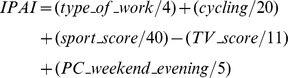



In order to classify the subjects into a particular activity level, IPAI score values were ascribed to five IPAI activity levels as described in the methods section.

We compared the correlation coefficients of the IPAI and the existing indices (Cambridge Index and Total Physical Activity Index) (Table 3). The IPAI showed moderate validity as continues, as well as categorical variable with r ≥ 0.4 for accelerometer counts and PAEE, and r>0.3 for MVPA and PAL. The older indices showed consistently weaker correlations.

**Table 3:** Spearman correlation coefficients (r) and 95% confidence intervals (95% CI) between accelerometer counts, Physical Activity Energy Expenditure (PAEE), Physical Activity Level (PAL), Moderate and Vigorous Physical Activity (MVPA), sedentary time, the Improved Physical Activity Index (IPAI) (continuous and in categories), the Cambridge Index, and the Total Physical Activity Index in 433 participants of the EPIC Germany study training sample.

Spearman Correlation Coefficients, n = 433					
		IPAI	IPAI categorical	Cambridge Index	Total PA Index
**Accelerometer**	**r**	0.43	0.44	0.29	−0.01
**counts (counts/min/day)**	**95%CI**	0.35,0.50	0.36,0.51	0.21,0.38	−0.11,0.08
**PAEE (kJ/kg/day)**	**r**	0.40	0.40	0.20	−0.05
	**95%CI**	0.32,0.48	0.32,0.48	0.11,0.29	−0.15,0.04
**PAL categories**	**r**	0.32	0.32	0.21	0.01
	**95%CI**	0.25,0.42	0.25,0.41	0.11,0.30	−0.08,0.10
**MVPA (min/day)**	**r**	0.34	0.34	0.25	0.04
	**95%CI**	0.24,0.40	0.24,0.41	0.16,0.34	−0.05,0.14
**Sedentary time**	**r**	−0.28	−0.27	−0.13	−0.00
**(min/day)**	**95%CI**	−0.36,−0.19	−0.36,−0.18	−0.22,−0.04	−0.10,0.09

Finally, we calculated the IPAI in the validation sample of 911 participants and again compared the obtained results with existing indices (Table 4). The correlation coefficients between the IPAI and accelerometer counts and PAEE decreased compared to the training sample (from r = 0.40−0.44 to r = 0.32−0.39) but still showed stronger correlations than the older indices. The correlation coefficients between MVPA, PAL, sedentary time and the IPAI were similar to the correlations coefficients for the Cambridge Index. No significant correlations with the objective measures of PA could be found for the Total Physical Activity Index.

**Table 4:** Spearman correlation coefficients and 95% confidence intervals (95% CI) between accelerometer counts, Physical Activity Energy Expenditure (PAEE), Physical Activity Level (PAL), Moderate and Vigorous Physical Activity (MVPA), sedentary time, the Improved Physical Activity Index (IPAI) (continuous and in categories), the Cambridge Index, and the Total Physical Activity Index in 911 participants of the EPIC Germany study validation sample.

Spearman Correlation Coefficients, n = 911					
		IPAI	IPAI categorical	Cambridge Index	Total PA Index
**Accelerometer**	**r**	0.40	0.39	0.24	−0.04
**counts (counts/min/day)**	**95%CI**	0.35,0.46	0.33,0.44	0.18;0.30	−0.10,0.03
**PAEE**	**r**	0.33	0.32	0.22	0.02
**(kJ/kg/day)**	**95%CI**	0.28,0.39	0.26,0.38	0.16,0.29	−0.04,0.09
**PAL categories**	**r**	0.30	0.29	0.21	0.05
	**95%CI**	0.24,0.36	0.22,0.34	0.14,0.27	−0,01,0.12
**MVPA (min/day)**	**r**	0.28	0.26	0.24	0.05
	**95%CI**	0.22,0.34	0.20,0.32	0.18,0.30	−0.02,0.11
**Sedentary time**	**r**	−0.27	−0.26	−0.19	−0.06
**(min/day)**	**95%CI**	−0.33,−0.21	−0.32,−0.20	−0.25,−0.13	−0.13,0.00

The Spearman correlation results for the sensitivity analysis within the group of non-working participants are presented in Table 5. The coefficients for the IPAI and the Cambridge Index decreased compared to the full sample but not for the Total Physical Activity Index. There was still a moderate correlation between the objective PA measures and the IPAI (r = 0.25−0.34). The correlations between the IPAI and the PA measures were significantly higher than for the older indices.

**Table 5:** Spearman correlation coefficients and 95% confidence intervals (95% CI) between accelerometer counts, Physical Activity Energy Expenditure (PAEE), Physical Activity Level (PAL), Moderate and Vigorous Physical Activity (MVPA), sedentary time, the Improved Physical Activity Index (IPAI) (continuous and in categories), the Cambridge Index, and the Total Physical Activity Index in 699 non-working participants of the EPIC Germany sub-study.

Spearman Correlation Coefficients, n = 699					
		IPAI	IPAI categorical	Cambridge Index	Total PA Index
**Accelerometer**	**r**	0.34	0.33	0.20	0.08
**counts (counts/min/day)**	**95%CI**	0.28,0.41	0.27,0.40	0.13,0.27	0.01,0.16
**PAEE (kJ/kg/day)**	**r**	0.29	0.27	0.17	0.10
	**95%CI**	0.22,0.36	0.20,0.34	0.09,0.24	0.03,0.18
**MVPA (min/day)**	**r**	0.27	0.26	0.21	0.11
	**95%CI**	0.20,0.34	0.19,0.33	0.13,0.28	0.03,0.18
**PAL categories**	**r**	0.27	0.25	0.16	0.11
	**95%CI**	0.20,0.34	0.18,0.32	0.09,0.24	0.04,0.18
**Sedentary time**	**r**	−0.24	−0.22	−0.13	−0.09
**(min/day)**	**95%CI**	−0.30,−0.16	−0.29,−0.15	−0.20,−0.05	−0.17,−0.02

**Figure 1:**
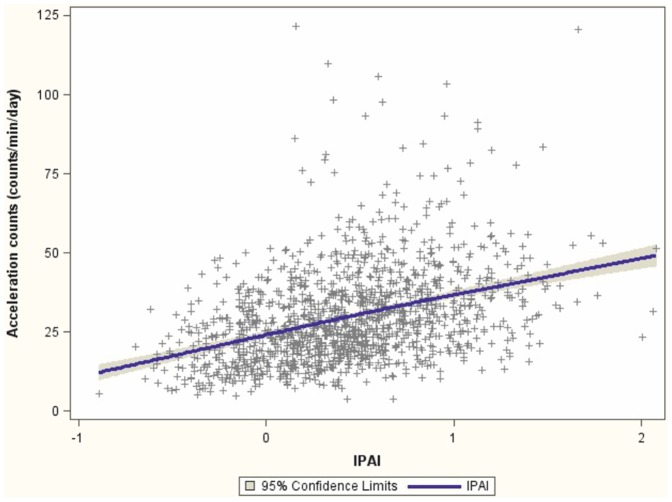
Linear regression analysis between accelerometer counts and the countinuously measured Improved Physical Activity Index (IPAI) in 1,344 participants of the EPIC Germany sub-study.

Finally, regression analysis revealed that a one point increase in the IPAI score is related to a 12.61 (standard error  = 0.84) increase in average accelerometer counts per minute (Figure 1). Based on the relationship between the IPAI score and the objectively measured accelerometer counts, error correction models can be performed in future analyses.

The distribution presented in Figure 1 shows that the 5^th^ percentile of the IPAI score (−0.30) equals an acceleration count value of 20.41 counts/min/day, the 25^th^ percentile (−0.12) an acceleration count of 25.69, the median IPAI (0.41) an accelerometer count of 29.35, the 75^th^ percentile (0.72) an acceleration count of 33.26, and the 95^th^ percentile (1.20) an acceleration count of 39.33.

## Discussion

The developed IPAI is a valid PA index that categorizes people according to their activity level and is able to express PA on a continuous scale. The index is linearly correlated with the participants' PA and sedentary time, and can be used similarly to acceleration counts. Furthermore, because activity at work is not the major contributor for the index, it is suitable for elderly populations or populations with a high proportion of non-working participants. Based on the performed regression analysis, risk estimate measurement error correction can be performed in studies using the index.

The main strengths of our study are a big study population of 1,344 participants and the objective PA measurement. The seven day heart rate and acceleration measurements have shown to be a valid method for measuring habitual PA [9]. The measurement included weekdays and weekends and therefore accounts for differences in weekly activity patterns [16]. The implemented extensive physical activity questionnaire included not only different timeframes but also secondary PA determinants like the living environment (neighborhood) and allowed us to deeply explore the associations with objectively measured PA and choose the most fitting pool of questions. The time-consuming questionnaire might have promoted errors during the filling in. Another limitation is seen in the objective PA measurement which might have introduced the Hawthorne effect, which implies that the participants change their behavior during the period they are observed [17]. A source of error in our PA measurement might be seasonal variability in performing PA. Two or more measurement time points, few months apart, would have been better to account for PA seasonality [16]. Finally, due to beta-blocker medication some participants were excluded from the step test which did not allow fitting an individual calibration model but a group calibration which might have introduced additional bias.

The IPAI showed moderate correlations with accelerometer counts (r = 0.40−0.43) and PAEE (r = 0.33−0.40) in the EPIC Germany population. A recent systematic review of 130 PA questionnaires showed that the median validity coefficients of existing (previously published results) and newly developed questionnaires are 0.30–0.39 and 0.25–0.41, respectively [1]. This places our IPAI among the top end of available questionnaire indices.

The Total Physical Activity Index surprisingly showed very low correlations with objectively measured PA. In the RPAQ validation study the Total Physical Activity Index showed weak correlations with PAEE of r = 0.14, 95% CI 0.04, 0.24 [3]. Nevertheless, in the study centers Netherlands and France, the correlations were negative or non-significant. The I^2^ = 80.5% in the center specific analysis showed high heterogeneity which might explain our results [3]. Moreover, the Total Physical Activity Index was developed and validated under different study conditions (first the index was developed for PA study purposes and the validation was performed later, on a different population) [13], [3]. In our study the Cambridge Index showed correlations comparable to that obtained by the validation study [3]. The first Cambridge Index validation was performed on a different study population than it was originally developed for [12]. In contrast, the IPAI development and evaluation were performed under highly similar study conditions. Therefore, the differences in index performance between the IPAI and the established indices might be also affected by differences in used study populations, countries, recruitment strategies and measurement software.

The EPIC study used the RPAQ and the EPIC-PAQ for assessing PA. The Cambridge Index showed moderate validity in our study as well as in the RPAQ validation study [3]. Nevertheless, after exclusion of employed participants the Spearman correlation decreased to r = 0.20 for accelerometer counts and r = 0.17 for PAEE. The correlation coefficient of the IPAI categories also decreased but showed still a moderate correlation of r = 0.34 for accelerometer counts and r = 0.29 for PAEE. In the German EPIC study already almost 50% of the participants are not working anymore. Especially for this population the IPAI represents a good alternative for categorizing elderly and not actively employed participants, into activity levels and more importantly, not misclassifying their activity level. So far, not many PA questionnaires have been validated in a similar population with emphasis on non-working participants. Elderly populations are of increasing interest and other strategies, than occupational activity cross-classification, have to be considered. The International Physical Activity Questionnaire (IPAQ) in its long and short form is one of the few questionnaires validated in this age group (correlation with acceleration counts MET/min/week r = 0.42 for men and r = 0.49 for women) [18], [19].

One of the variables included in the index was weekend evening computer use. Surprisingly, this variable representing sedentary activity was positively correlated with objectively measured PA. It is possible that weekend evening computer use is a proxy measure for being active during the weekdays or for socio-economic PA determinants like educational attainment which has been shown to be positively correlated with maintaining a healthy lifestyle [20] and being physically active [21].

Acceleration counts is an accelerometer output which might differ by accelerometer type and its settings [22]. For the accelerometer used in the Actiheart device, the mean for accelerometer counts was 29.5 in our study which is approximately more than 600 kcal/day of PAEE. Based on this knowledge, and the relationship between accelerometer counts and the IPAI (β), future research that uses the IPAI will be able to take into account the measurement error. This enables measurement error correction in future studies using the index [15], [23].

## Conclusions

In conclusion, we succeeded in developing a valid physical activity index, which is able to categorize people into activity categories and can be used similarly to accelerometer counts. We propose the use of the IPAI in future EPIC-Germany follow-up surveys because in populations with a high rate of non-working participants (like in EPIC) the performance of the IPAI is significantly better than the established indices. A validation in other EPIC countries would be of particular interest.
